# Surgical correction of ectopic penis and scrotum associated with bilateral orchidopexy

**DOI:** 10.1590/S1679-45082017RC3927

**Published:** 2017

**Authors:** Daniel Santos Rocha Sobral, Helder Damásio da Silva, Eulálio Damázio

**Affiliations:** 1 Universidade Federal do Piauí, Teresina, PI, Brazil.; 2 Universidade Federal de São Paulo, São Paulo, SP, Brazil.

**Keywords:** Congenital abnormalities, Penis, Scrotum, Cryptorchidism, Orchiopexy, Case reports

## Abstract

Ectopic penis is usually associated with penoscrotal transposition, and it is rarely observed in isolation. We report a surgical approach for an extremely rare case. A 10-year-old male patient with bilateral cryptorchidism and ectopic penis and scrotum in perineal area, with no penoscrotal transposition, representing an association not yet described in literature. A previous orchiopexy failed due to ectopic scrotum. By means of an inverted Y incision, the penis was mobilized and a perineal skin flap in form of a testicular sac was prepared. Finally orchiopexy was performed. The surgery was essential to treat cryptorchidism and to improve the self-image of the patient.

## RESUMO

O pênis ectópico geralmente ocorre associado à transposição peno-escrotal, sendo raro isoladamente. Relatamos uma abordagem cirúrgica para um caso extremamente raro. Tratava-se de paciente do sexo masculino, 10 anos, com criptorquidia bilateral e pênis e escroto ectópicos, na região perineal, sem transposição peno-escrotal, representando uma associação ainda não descrita na literatura. Orquidopexia prévia sem sucesso, devido à ectopia do escroto. Por meio de uma incisão em Y invertido, mobilizou-se o pênis e preparou-se um retalho da pele perineal em forma de bolsa testicular. Por fim, realizou-se a orquidopexia. A cirurgia foi fundamental para tratar a criptorquidia e promover ganho na autoimagem do paciente.

## INTRODUCTION

Several congenital genital anomalies affect males, and cryptorchidism is the most frequent disorder.^1^ Other conditions are rare, including ectopic scrotum and ectopic penis; the latter is more often reported in cases of penoscrotal transposition.^2^ We report a completely new surgical approach for an extremely rare case, due to association of ectopic penis, ectopic scrotum and bilateral cryptorchidism.

## CASE REPORT

**Figure 1:**
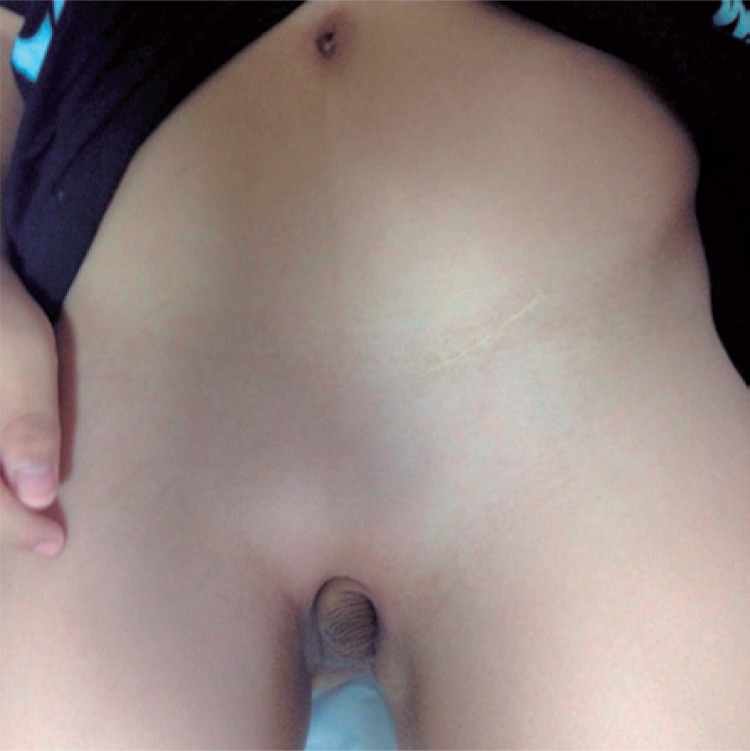
Ectopic penis and scrotum located in the perineal region

**Figure 2:**
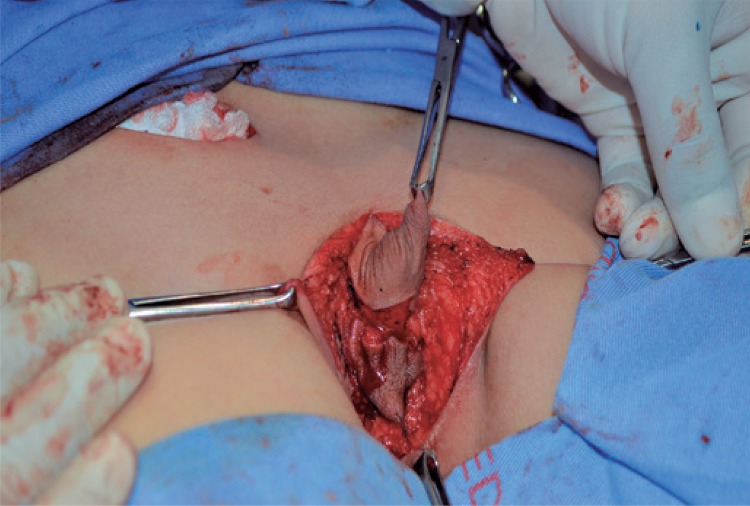
Inverted Y incision, complemented inferiorly to separate the penis from the scrotum

**Figure 3:**
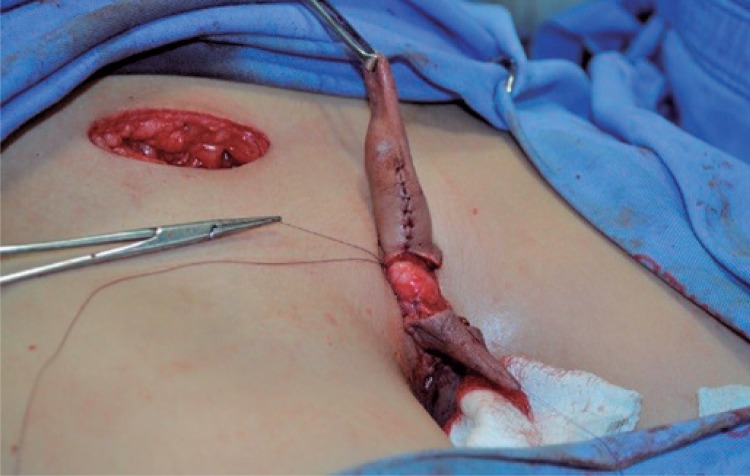
Positioning the right testis in the right hemiscrotum sac, formed with perineal scrotal skin

A 10-year-old male patient with ectopic penis and scrotum (Figure 1) in the perineal region, with bilateral cryptorchidism and without penoscrotal transposition. Moreover, he presented with skeletal deformity, including pubic diaphysis and ankylosed knees, and no associated urinary tract anomalies. The child had been previously submitted to unsuccessful left orchiopexy due to ectopic scrotum; the testis was positioned in the base of the penis. An inguinal incision was first performed on the right side to mobilize the right testis. However, it was possible to move it up to the anatomical position of the scrotum, in the pubis, and not to the ectopic position, in the perineal region. Next, the surgical correction of the ectopic penis and scrotum was performed, initiating with an inverted Y incision, complemented inferiorly to separate the penis from the scrotum (Figure 2). The penile was later moved from the perineum to its anatomical position in the pubis, where it was fixated. A perineal scrotal skin flap was used to prepare the scrotal sac, adjacent and inferiorly to the penis. The median raphe and two hemiscrotum sacs were formed. The next step was orchiopexy, initiated by positioning the right testis in the right hemiscrotum sac (Figure 3), and continued by approaching the left testis up to its placement in the left hemiscrotum sac. Finally, circumcision was performed.

**Figure 4:**
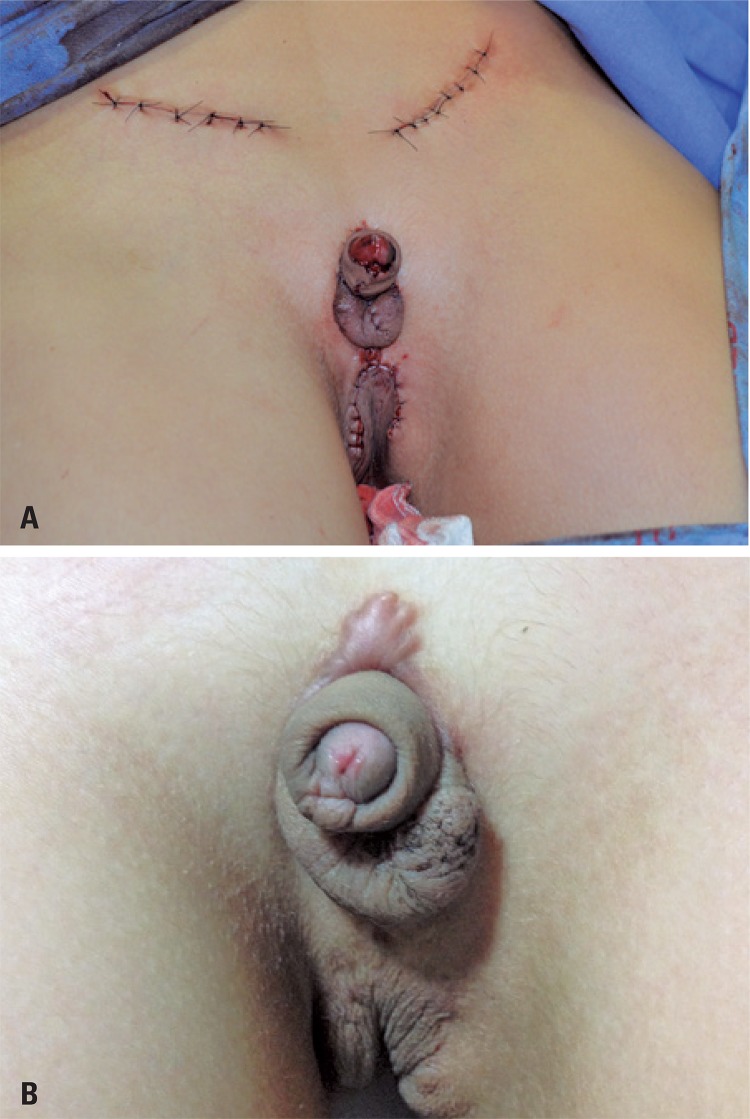
Immediate postoperative period (A) and follow-up visit 8 months later (B) show the successful surgical result

In the immediate postoperative period (Figure 4A), we could observe the placement of the penis and scrotum in their anatomical positions, with the testis accommodated in their respective hemiscrotum sacs. The perineal skin had no tension at all due to non-excessive removal of scrotal and perineal skin. Eight months after surgery (Figure 4B), there was good healing, the perineal skin had no tension, and all structures were kept in their topical positions, preserving their functions and with no complications.

## DISCUSSION

Ectopic penis is an anomaly usually associated with penoscrotal transposition. As an isolated abnormality, there are less than 20 cases described in the literature.^3^ Ectopic scrotum is the rarest anomaly of this gland.^4^ In the patient herein reported, the ectopic penis and scrotum represented an association not described in the literature yet. Although ectopic, these structures maintained their position relative to each other, but not characterizing a penoscrotal transposition.^5^ The anomaly of this patient is probably a result of bone malformation at the level of the pubic diaphysis. The rare association and absence of reports justify the late surgical repair of ectopy. It was essential to treat cryptorchidism in this case, as demonstrated by the unsuccessful attempt to perform orchiopexy before correcting the ectopic positions. In addition to enabling orchiopexy, the surgical procedure led to improved self-image of the patient.
